# A Meta-Analysis of Apolipoprotein E Gene ε2/ε3/ε4 Polymorphism for Gallbladder Stone Disease

**DOI:** 10.1371/journal.pone.0045849

**Published:** 2012-09-25

**Authors:** Pei Xue, Wen-Quan Niu, Zhao-Yan Jiang, Min-Hua Zheng, Jian Fei

**Affiliations:** 1 Department of Surgery, Ruijin Hospital, Shanghai Jiao Tong University School of Medicine, Shanghai, China; 2 Shanghai Institute of Digestive Surgery, Shanghai, China; 3 Shanghai Institute of Hypertension, Shanghai, China; 4 Shanghai Minimally Invasive Surgery Center, Ruijin Hospital, Shanghai Jiao Tong University School of Medicine, Shanghai, China; University of Colorado, United States of America

## Abstract

**Background:**

Numerous studies have investigated the relationship between apolipoprotein (Apo) E gene polymorphisms and gallbladder stone disease (GSD) across ethnic populations; however, the results are often inconsistent. This meta-analysis aims to comprehensively evaluate the influence of a common ε2/ε3/ε4 polymorphism in Apo E gene on the risk of gallbladder stone disease.

**Method:**

Data were analyzed using the RevMan software (V5.1) and a random-effects model was applied irrespective of between-study heterogeneity. Publication bias was weighed using the fail-safe number.

**Results:**

There were 17 study populations totaling 1773 cases and 2751 controls for ε2/ε3/ε4 polymorphism of Apo E gene. Overall comparison of alleles ε2 with ε3 in all study populations yielded a 16% decreased risk for GSD (95% confidence interval [95% CI]: 0.68–1.05; P = 0.31; *I^2^* = 13%), and comparison of alleles ε4 with ε3 yielded a 25% increased risk (95% confidence interval [95% CI]: 0.97–1.61; P = 0.0003; *I^2^* = 63%). Subgroup analysis by study design indicated that the magnitude of association in hospital-based studies was largely significantly strengthened for ε4 allelic model (odds ratio [OR]  = 1.46; 95% CI: 1.05–2.02; p = 0.0007; *I^2^* = 65%). Subgroup analysis by age of controls indicated a remarkably significant elevation in the magnitude of association in age >50 subgroups in ε4 allelic model (OR = 1.50; 95% CI: 1.03–2.19; p = 0.0009; *I^2^* = 72%). Moreover, subgroup analysis by cases gender indicated a reduction in the magnitude of association in male<30% studies for E2/2 genotypic model (OR = 0.32; 95% CI: 0.07–1.49; p = 0.16; *I^2^* = 45%).

**Conclusions:**

Our results reveal that Apo E gene ε4 allele is a risk factor of gallbladder stone disease, especially in elder people and Chinese population.

## Introduction

Gallbladder stone disease (GSD) is one of the most common digestive disorders worldwide, especially in western population [1]. In China, the incidence of GSD is gradually increasing and becomes a public health problem with high economic burden. The prevalence of gallstone disease is extremely higher in certain ethnic groups such as Pima Indians suggesting possible genetic factors involved in the pathogenesis of GSD. Recently, the Swedish twin study strongly indicates that genetic factors play a role in gallstone formation [2].

One of the candidate lithogenic-genes had been studied is apolipoprotein (Apo) E. It is a ligand for the low density lipoprotein family of receptors and plays a pivotal role in cholesterol metabolism [3], [4]. Apo E has three common isoforms, ε2, ε3, and ε4, respectively, at a single locus in chromosomal region 19q13.2. These alleles define six Apo E phenotypes: E2/2, E3/3, E2/4, E3/3, E4/3, and E4/4. A large number of individually underpowered studies have been conducted on Apo E polymorphisms across different ethnic populations. However, the results are somewhat irreproducible and inconclusive. In this meta-analysis, we aimed to evaluate the association between different genotypes of Apo E with GSD using data from different countries, while addressing between-study heterogeneity and publication bias.

## Methods

### Literature Search

Publication were searched via public database PubMed (http://www.ncbi.nlm.nih.gov/pubmed/), Embase (http://www.embase.com), ISI Wed of Knowledge (http://isiknowledge.com), Wanfang (http://www.wanfangdata.com.cn) and China Biological Medicine (CBM) (http://cbm.imicams.ac.cn) with the last update as of June 2012. The keywords used for search were ‘gallbladder stone disease’ and ‘Apolipoprotein E or Apo E’ combined with ‘gene or variants or polymorphism or alleles’, all of which were MeSH terms (Medical Subject Headings in the US National Library of Medicine). The ‘related articles’ option in MEDLINE, as well as reference lists of all retrieved studies, were checked to search for other relevant publications that were not initially identified. As a prerequisite, only these published in English or Chinese language were identified, and studies in human subjects. In addition, the full text of the retrieved articles was scrutinized to make sure the data of interest were included. If two or more studies shared the same cases or control subjects, the one with small sample size was abandoned. If more than one geographical or ethnic population were included in one article, each population was considered separately.

**Figure 1:**
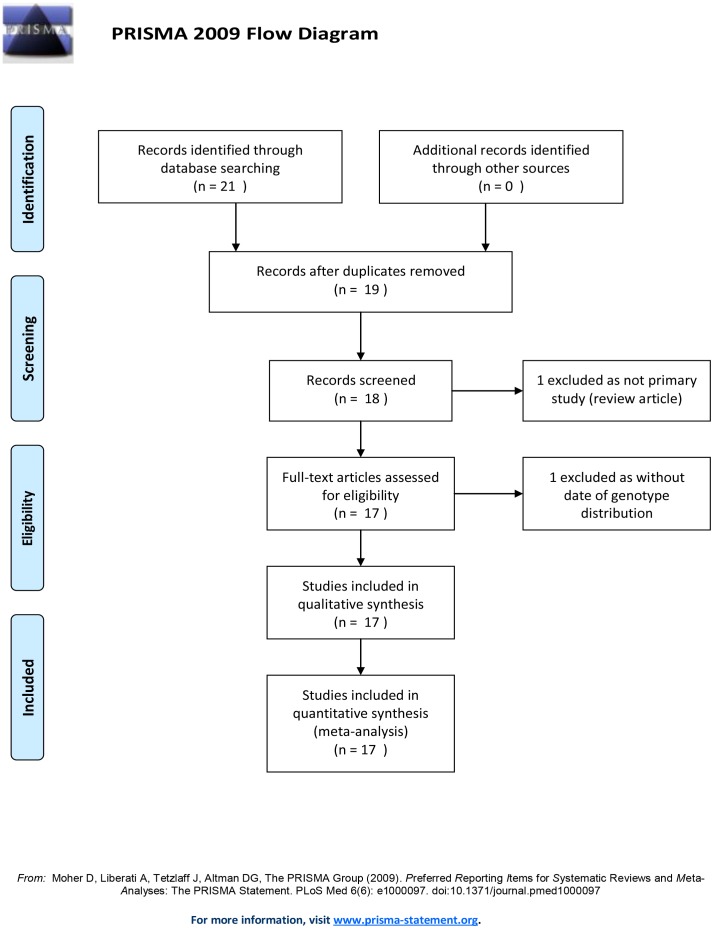
Flow diagram of search strategy and study selection.

### Inclusion/Exclusion Criteria

Studies that we identified satisfied the following criteria: 1. evaluation of Apo E polymorphism in association with gallbladder stone disease; 2. case-control study using either a hospital-based or a population-based design; 3. genotype/allele counts of Apo E polymorphisms between cases and controls for estimating odds ratio (OR) and 95% confidence interval (95% CI).

Gallbladder stone disease was diagnosed by operation or ultrasound. In some studies, the cholesterol content in gallstone was analyzed. At the same time, some GSD with T2DM, hypertension, pregnancy, or underwent bariatric surgery were also included.

Studies were excluded if they were prospective studies; published in minor language; or published abstracts from meeting.

**Figure 2:**
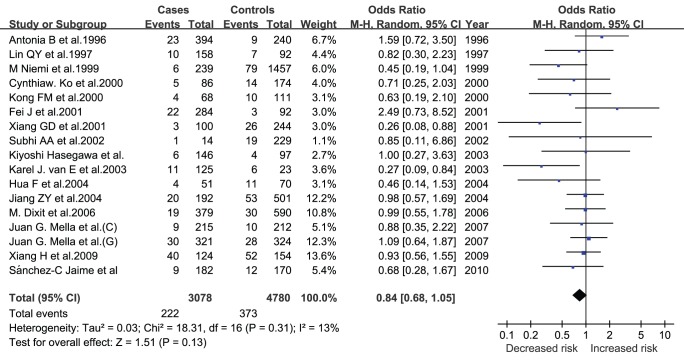
Pooled random-effects-based odds ratio of developing GSD for ε2 versus ε3.

**Table 1:** Comparisons of E2 vs E3 in allele, genotype dominant and recessive models for GSD risk.

Comparisons	Pooled OR (95% CI)	Z (P)	I^∧^2(%)
ε2 vs ε3	0.84 (0.68, 1.05)	1.51 (0.13)	13
E2/2 vs E3/3	0.49 (0.19, 1.25)	1.50 (0.13)	0
E2/3 vs E3/3	0.93 (0.73, 1.19)	0.57 (0.57)	0
E2/2+E2/3 vs E3/3	0.88 (0.69, 1.13)	1.00 (0.32)	0
E2/2 vs E2/3+E3/3	0.51 (0.20, 1.29)	1.43 (0.15)	0

Abbreviations: OR, odds ratio; CI, confidence interval.

### Extracted Information

Two authors (P. Xue and WQ. Niu) independently drew the following information from all qualified studies: first author’s last name, publication date, population ethnicity, methods to diagnosis of gallstone, study design, methods of genotyping, the distribution of alleles and the genotype in cases and controls. Information such as cases and controls’ age, BMI, and serum concentration of TC, TG, LDL, HDL were also collected. The units of measurements used in this study were transformed into the standard measurement units.

**Figure 3:**
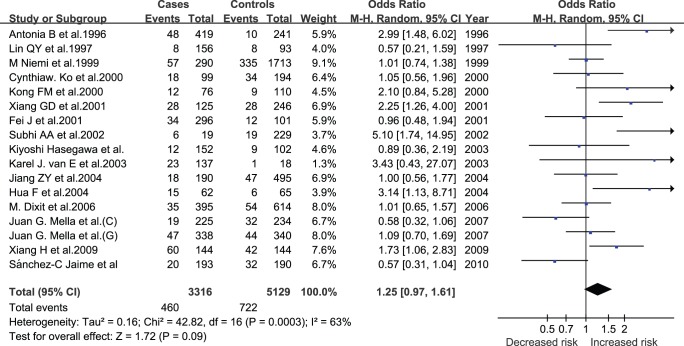
Pooled random-effects-based odds ratio of developing GSD for ε4 versus ε3.

**Table 2:** Comparisons of E4 vs E3 in allele, genotype dominant and recessive models for GSD risk.

Comparisons	Pooled OR (95% CI)	Z (P)	I^∧^2(%)
ε4 vs ε3	1.25 (0.97, 1.61)	1.72 (0.09)	63
E4/4 vs E3/3	1.53 (0.87, 2.69)	1.49 (0.14)	0
E3/4 vs E3/3	1.33 (0.94, 1.89)	1.59 (0.11)	70
E3/4+E4/4 vs E3/3	1.38 (0.97, 1.96)	1.80 (0.07)	71
E4/4 vs E3/4+E3/3	1.44 (0.82, 2.52)	1.27 (0.20)	0

Abbreviations: OR, odds ratio; CI, confidence interval.

### Statistical Analysis

The associations between genotypes/alleles of Apo E polymorphism with GSD were evaluated by using the software Review Manager (V5.1) for windows. In this meta-analysis, we used the random-effects model with the method of DerSimonian & Laird to bring the individual effect-size estimates together. The estimate of heterogeneity was taken from the Mantel-Haenszel model [5].

Heterogeneity was assessed by the *I^2^* statistic, which was documented for the percentage of the observed between-study variability due to heterogeneity rather than chance with the ranges of 0 to 100% [*I^2^* = 0–25%, no heterogeneity; *I^2^* = 25–50%, moderate heterogeneity; *I^2^* = 50–75%, large heterogeneity; *I^2^* = 75–100%, extreme heterogeneity] [6].

We assessed publication bias using the fail-safe number (N_fs_) with the significance set at 0.05 for each meta-comparison. Specifically, if the calculated N_fs_ value was smaller than the number of observed studies, than the meta-analysis results might run the risk of having publication bias. We calculated the N_fs_0.05 according to the formula N_fs_0.05 =  (ΣZ/1.64)^2^–k, where k is the number of included articles [7]–[9].

**Table 3:** Fail-safe number.

E2/2 versus E3/3	4.16
E2/3 versus E3/3	32.77
E2/4 versus E3/3	25.23
E3/4 versus E3/3	201.28
E4/4 versus E3/3	27.89
E2 versus E3	55.04
E4 versus E3	156.47

## Results

### Studies and Populations

Under the guide of our searching strategies, we identified 21 eligible articles (12 in English and 9 in Chinese) at the first time. According to our inclusion/exclusion criteria, we removed 5 of them for the following reasons: three papers shared same study group, one review, one without data of genotype distribution. Sixteen qualified studies (10 in English [2] [10] [11]–[18], 17 populations) totaling 1773 gallbladder stone disease patients and 2751 controls were further analyzed. Basic characteristics of study populations are presented in Table S1. A diagram schematizing the selection process of identified studies is presented in Figure 1.

### Pooled Analysis

As for Apo E, in allelic model, comparison of ε2 with ε3 allele generated a non-significant 16% decreased GSD risk (P = 0.31) (Figure 2). No significance was observed in genotypic models for comparisons of E2/2 (OR = 0.49; P = 0.82) and E2/3 (OR = 0.93; P = 0.83) genotypes with E3/3 genotype, respectively, as well as in dominant (OR = 0.88; P = 0.70) and recessive (OR = 0.51; P = 0.81) models. The *I^2^* statistic indicated no between-study heterogeneity except for that of ε2 with ε3 allele (*I^2^* = 13%). (Table 1).

The comparison of ε4 with ε3 allele generate a significant 25% increased risk (P = 0.0003) (Figure 3). There was also significance in genotypic models for the comparisons of E3/4 with E3/3 genotype (OR = 1.33; P<0.00001). No significance was observed in the comparison of E4/4 with E3/3 genotype (OR = 1.53; P = 0.80). Furthermore, a significant increased risk was present in dominant models (OR = 1.38; P<0.0001), but not in recessive models (OR = 1.44; P = 0.91). The *I^2^* statistic indicated the large between-study heterogeneity for all comparisons except for that of E4/4 with E3/3 (*I^2^* = 0) and recessive models (*I^2^* = 0). (Table 2).

Moreover, since most of studies were conducted in Chinese *Han* population, after restricting analyses to Han populations, there was no material change in OR for both polymorphisms under study (data not shown).

### Subgroup Analysis

Considering the fact that study design, samples age, gender distribution or ethnic differences might bias the overall association, we conducted separate analysis according to these factors.

In view of study design, the magnitude of association in population-based studies was weakened for ε2 allelic model (OR = 0.91; 95% CI: 0.52–1.59; p = 0.15), ε4 allelic model (OR = 0.92; 95% CI: 0.73–1.16; p = 0.45), E3/4 genotypic model (OR = 0.80; 95% CI: 0.52–1.23; p = 0.14) and E4/4 genotypic model (OR = 0.78; 95% CI: 0.33–1.89; p = 0.95). Meanwhile, the magnitude of association in hospital-based studies was largely significantly strengthened for ε4 allelic model (OR = 1.46; 95% CI: 1.05–2.02; p = 0.0007), E3/4 genotypic model (OR = 1.69; 95% CI: 1.07–2.67; p<0.0001), E4/4 genotypic model (OR = 2.45; 95% CI: 1.18–5.09; p = 0.87), ε2 recessive model (OR = 0.39; 95% CI: 0.13–1.18; p = 0.69) and ε4 dominant model (OR = 1.77; 95% CI: 1.12–2.80; p<0.0001). Nearly no change in ORs was observed in other subgroup analysis for study design.

The age of onset of GSD differed from different studies. Since GSD prevalence increases with age, to avoid the miss-classification, we choose age of 50 as the cut-off in controls to divide the studies into two subgroups. A remarkable significantly strengthen in the magnitude of association in age >50 subgroups in ε4 allelic model (OR = 1.50; 95% CI: 1.03–2.19; p = 0.0009), E3/4 genotypic model (OR = 1.90; 95% CI: 1.14–3.16; p = 0.0003) and ε4 dominant model (OR = 1.99; 95% CI: 1.16–3.40; p<0.0001) could be observed. No significant change in ε2 models or other subgroups was present.

The ratio of GSD in female and male is about 2∶1, thus we choose the 30% gender percentage (males in cases) as the cut-off to divide populations into two subgroups, considering that the number of each subgroup is almost equal at the same time. We found that the magnitude of association in male <30% studies was strengthened for E2/2 genotypic model (OR = 0.32; 95% CI: 0.07–1.49; p = 0.16) and the magnitude of association in male >30% studies was strengthened for E4/4 genotypic model (OR = 1.92; 95% CI: 0.81–4.53; p = 0.31). This suggests that ε2 may provide protection against GSD in women, and on contrary, ε4 is a risk gene promoting GSD. Although the prior viewpoint has been proposed by Niemi et al in 1999 [10], this result didn’t get strong support from other subgroup analysis in our meta-analysis.

In order to control for the difference of ethnicity, we separated the studies in Chinese and non-Chinese. The magnitude of association in Chinese studies was significantly strengthened for both ε4 allelic model (OR = 1.47; 95% CI: 1.01–2.13; p = 0.07) and ε4 dominant model (OR = 1.88; 95% CI: 0.97–3.62; p = 0.002). There was no significant change in other subgroups.

The result of publication bias assessed by the fail-safe number (N_fs_) was shown in Table 3.

## Discussion

In this meta-analysis, we used 17 study populations (a total of 4524 subjects, from 16 publications) to evaluate the association of Apo E gene polymorphisms with GSD. To the authors’ knowledge, this is the first meta-analysis investigating the association between Apo E ε2/ε3/ε4 polymorphisms and GSD. Although some statistical bias could not be eliminated, this meta-analysis suggests that the Apo E ε4 allele appears to be associated with an increased risk of GSD and also appears to bedominant.

Previous researches have shown that the presence of the ε4 allele of Apo E is strongly associated with the risk of atherosclerosis [19] and Alzheimer’s disease [20]. Our present meta-analysis indicates that Apo ε4 allele is a risk factor for the development of gallstones. Due to different affinity to its receptors, Apo E can eventually influence hepatic cholesterol processing by enhancing cholesteryl ester hydrolysis [21], thus increasing cellular free cholesterol availibity for biliary secretion. There’s also evidence that Apo ε4 leads to more intracellular release of free cholesterol from internalized triglyceride-rich particle cholesteryl ester than does ε3 [22]. Our meta-analysis give us a clear conclusion that ε4 allele is a risk factor of gallbladder stone disease, especially in elder people and Chinese people. In Chinese studies, the result that ε4 is a risk factor seems more obvious, although this result is not supported by every Chinese study. We noticed that 7 of 16 qualified studies are published in Chinese in this meta-analysis. Thus, ethnic diversity may affect the process of gallstone formation. To insure the validity of the overall conclusions, we divided the studies into Chinese and non-Chinese. There’s no significant change for the association of ε4 allelic model and ε4 dominant model, though the magnitude was strengthened for Chinese. Due to the limitation of the number of studies published, our analysis would not be able to include all ethnicities so far. However, we could not exclude the possibility that some negative results may not have chance to get published and would in certain extent overestimate the results.

Actually, in the newest review by Laura M. Stinton and Eldon A. Shaffer [23], they think that geography and particularly ethnicity play an enormous role in the prevalence of gallstone disease and also the type of stone that forms: cholesterol gallstones predominate in the developed countries of the Western world; brown pigment stones in the bile ducts are more common in Asia.

The prevalence of gallstone disease increases with age, escalating markedly after middle age, becoming 4–10 times more likely in older individuals [24]. The age of cases and controls in the studies we collected ranged from 20 to 70, so we divided them into two subgroups as to discover the influence related with age. In our study, we chose 50 years of age as the cut-off for controls. A similar approach has been used by other investigators [25]. If we selected a lower age, the number of studies in the two subgroups would be unbalanced and cause possible statistical migration. Thus, we decided to use 50 years as the cut-off to assure the number of studies in the two subgroups were as equal as possible. Fortunately, we can seize a more significant result from elder subgroups about ε4 allele, which is consistent with natural law.

By contrast, the conclusion of ε2 allele is equivocal. It seems that ε2 allele can provide a protection against GSD in women. Nimei [10] et al. proposed that the protective effect of ε2 may lie in the metabolic pathways leading to supersaturation, as subjects with the ε2 allele show low cholesterol absorption and a high rate of bile salt synthesis [26]. Moreover, it seems that gender also play in part a role on ε2 allele and affect the susceptibility to gallstone formation.

Last but not least, subgroup analysis by study design showed that hospital-based studies yielded a more significant association signals than population-based studies. Generally, in population-based studies, it is not clear if the people in controls had other diseases which could exert a confounding effect on the true association. Only 4 publications were population-based study, it is still too little for the meta-analysis. Thus, the results on study design should be more cautious for interpretation.

Despite the clear strength of our study including large sample sizes, some limitations merit serious consideration. First, all included studies were cross-sectional design, which precludes further comments on cause-effect relationship [27]. Second, most studies have recruited subjects aged>40 years, for whom environmental factors are likely to contribute more prominently than the genetic component during the development of GSD. Thus, large association studies in a younger population of GSD subjects are of added interest. Third, due to the relative small number of some studies, we were unable to perform further subgroup analyses such as by gender and age. Fourth, in this study, we only focused on Apo E gene polymorphisms, and were not able evaluate other genes or polymorphisms responsible for GSD. It is possible that the potential role of genes such as ABCG5-G8 polymorphism [28] is diluted or masked by other gene-gene or gene environment interactions. Thus, the jury must refrain from drawing a conclusion until a large, well-performed worldwide study confirms or refuses our results.

Taken together, in this meta-analysis, we ascertained the role of Apo ε4 allele on the risk factor of GSD. Although the publication bias was maximally avoided, presence of between-study heterogeneity could not be fully explained by our subgroup analysis. Nowadays further analyses are warranted to investigate Apo E gene adjacent markers in a wider context, future studies on Apo E and GSD should concentrate on gene-environment interactions.

## Supporting Information

Table S1 (XLS)
